# Metastatic extent predicts survival as patients with metastatic castration-resistant prostate cancer are treated with ^177^Lu-PSMA radioligand therapy

**DOI:** 10.7150/thno.44568

**Published:** 2020-03-30

**Authors:** Finn Edler von Eyben, Harshad R. Kulkarni, Richard P. Baum

**Affiliations:** 1 Center of Tobacco Control Research, Odense, Denmark;; 2 Theranostics Center for Molecular Radiotherapy and Molecular Imaging, Zentralklinik Bad Berka, Bad Berka, Germany.

**Keywords:** prostate specific membrane antigen, ^17^7Lutetium [^177^Lu]-PSMA radioligand therapy, prostatic neoplasms, restaging, overall survival

## Abstract

PSMA based radioligand is a new investigational drug for treatment of metastatic multidrug-resistant and castration-resistant prostate cancer. Prognostic factors point to above and below average overall survival (OS) after the treatment.

Kessel et al. [Theranostics 2019;9:4841-8] reported for the first time that two sites of visceral metastases, lungs and liver, differed in impact on OS after treatment with ^177^Lu PSMA 617. Treatment with established drugs showed the same trend. The difference in OS between the sites is independent of the type of treatment and can reflect changes in tumor biology during the progression of metastatic prostate cancer.

The publication by Kessel et al. on ^177^Lu prostate specific membrane antigen (PSMA) radioligand therapy (PRLT) for patients with metastatic multidrug-resistant and castration-resistant prostate cancer (mCRPC) is interesting 1. Previous publications found visceral metastases had negative impact on overall survival (OS) after PRLT 2-5, whereas the Kessel publication evaluated whether PRLT for patients with bone, lung, and liver metastases differed in OS. The authors found that patients with or without lung metastases did not differ in OS.

In contrast, patients with liver metastases had a worse OS than patients without liver metastases. The findings pointed to a heterogeneity between patients with lung and liver metastases. In multivariate analyses, visceral metastases, both lung and liver metastases, had a significant negative impact on OS.

As an external validation of the findings, another German center, the Zentralklinik Bad Berka (ZBB), evaluated 174 consecutive patients. The OS is shown in Figure 1. Twenty-eight patients had lymph node metastases (LNM) with or without one or two bone metastases (site 1 and 2 in the figure), 102 had multiple bone metastases (site 3), 21 had lung metastases (site 4), and 23 had liver metastases (site 5). Treatment with ^177^LuPRLT included median 3 cycles, median 6.3 GBq per cycle, and median 8 weeks intervals between the cycles 6.

Of the patients, those with bone and lung metastases had grossly similar OS whereas the patients with liver metastases had a worse OS. In addition, patients with only LNM with or without one or two bone lesions had the best OS. The five patient groups differed markedly in OS (p < 0.0001, log rank test). The findings confirmed those of Kessel et al. The groups of patients treated at ZBB had a trend for OS that corroborated with that for the groups of patient Kessel et al. reported.

ZBB first reported that patients with LNM had a favorable outcome after PRLT in 2016 7. A recent paper of 45 patients with LNM confirmed that PRLT resulted in an impressive long OS for these patients 8. Also a systematic review of nine trials of patients with mCRPC treated with docetaxel found that patients with LNM had a better OS than those with bone, lung, and liver metastases 9. The review summarized more than 8000 patients. Patients with liver metastases treated with docetaxel had the worst OS.

Since nine trials of docetaxel as first-line treatment of mCRPC and two separate studies of PRLT as last-line treatment show the same trend for OS, it can be concluded that the sites of the progressing metastases of mCRPC differ in tumor biology. Kessel et al. discussed further that genetic changes in the progression of the metastases may contribute to the reduced outcome after treatment of the most advanced metastases. This idea is indeed thought-provoking.

Evaluated patients with bone, lung, and liver metastases had similar OS whether the patients had been treated with first-line docetaxel or last-line ^177^ LuPRLT, Therefore, we extrapolated that patients with LNM would have had a similar OS whether treated with docetaxel or PRLT. But, interestingly for patients with LNM, PRLT resulted in a better OS than docetaxel. The discrepancy suggests that for these patients PRLT has a significant effect on OS which cannot be ascribed only to a lead time phenomenon.

Guidelines from the Prostate Cancer Clinical Trials Working Group 3 (PCWG3) on reporting of trials of mCRPC laid emphasis on restaging with conventional imaging modalities such as CT and bone scans 10. But PSMA PET/CT is more sensitive and is increasingly used worldwide in the daily routine 11, 12. For patients with multiresistant mCRPC, a positive PSMA PET/CT is mandatory for initiation of PRLT. But today most patients with progressive prostate cancer and a positive PSMA PET/CT are not treated with PRLT.

Another recent analysis showed that also asymptomatic patients with mCRPC treated with PRLT had a two-year OS of 100%, clearly above the median OS of 13 months generally observed for PRLT of patients with multi-resistant mCRPC 13. Thus findings of retrospective studies such as that by Kessel et al. help to design new prospective controlled randomized trials to maximize the effect as patients with prostate cancer are treated with PRLT.

## Figures and Tables

**Figure 1:**
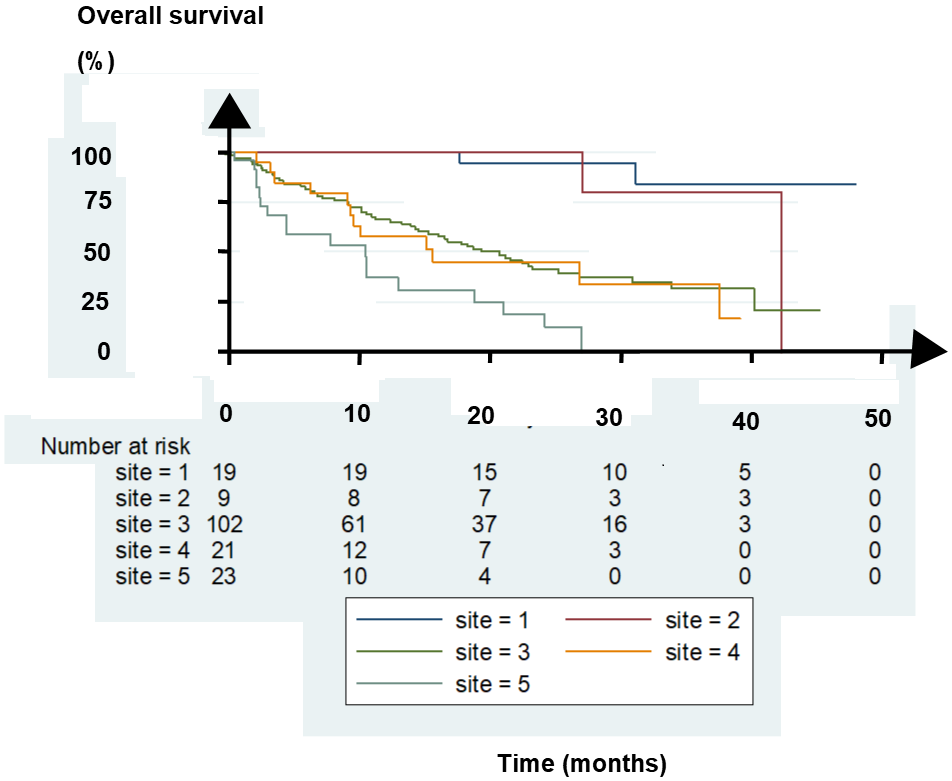
Kaplan-Meier plot of overall survival for patients with lymph node metastases (LNM) (site 1), LNM with one or two bone metastases, (site 2), bone metastases (site 3), lung metastases (site 4), and liver metastases (site 5).

## Abbreviations

LNM: lymph node metastases
mCRPC: metastatic castration-resistant prostate cancer
OS: overall survival
PRLT: PSMA radioligand therapy
PSMA: prostate specific membrane antigen
PCWG3: Prostate Cancer Clinical Trials Working Group 3

## References

[1] Kessel K, Seifert R, Schafers M, Weckesser M, Schlack K, Boegemann M (2019). Second line chemotherapy and visceral metastases are associated with poor survival in patients with mCRPC receiving (177)Lu-PSMA-617. Theranostics.

[2] Ahmadzadehfar H, Schlolaut S, Fimmers R, Yordanova A, Hirzebruch S, Schlenkhoff C (2017). Predictors of overall survival in metastatic castration-resistant prostate cancer patients receiving [(177)Lu]Lu-PSMA-617 radioligand therapy. Oncotarget.

[3] Heck MM, Tauber R, Schwaiger S, Retz M, D'Alessandria C, Maurer T (2019). Treatment Outcome, Toxicity, and Predictive Factors for Radioligand Therapy with (177)Lu-PSMA-I&T in Metastatic Castration-resistant Prostate Cancer. Eur Urol.

[4] Sathekge M, Bruchertseifer F, Vorster M, Lawal I, Knoesen O, Mahapane J (2020). Predicors of Overall and Disease free survival in Metastatic Castration resistant Prostate Cancer Patients Receiving (225)Ac-PSMA-617 Radioligand therapy. J Nucl Med.

[5] Yadav MP, Ballal S, Bal C, Sahoo RK, Damle NA, Tripathi M (2020). Efficacy and Safety of 177Lu-PSMA-617 Radioligand Therapy in Metastatic Castration-Resistant Prostate Cancer Patients. Clin Nucl Med.

[6] Barber TW, Singh A, Kulkarni HR, Niepsch K, Billah B, Baum RP (2019). Clinical outcomes of (177)Lu-PSMA radioligand therapy in taxane chemotherapy pretreated and taxane chemotherapy naive patients with metastatic castration resistant prostate cancer. J Nucl Med.

[7] Kulkarni HR, Singh A, Schuchardt C, Niepsch K, Sayeg M, Leshch Y (2016). PSMA-Based Radioligand Therapy for Metastatic Castration-Resistant Prostate Cancer: The Bad Berka Experience Since 2013. J Nucl Med.

[8] Edler von Eyben F, Singh A, Zhang J, Nipsch K, Meyrick D, Lenzo N (2019). (177)Lu-PSMA radioligand therapy of predominant lymph node metastatic prostate cancer. Oncotarget.

[9] Halabi S, Kelly WK, Ma H, Zhou H, Solomon NC, Fizazi K (2016). Meta-Analysis Evaluating the Impact of Site of Metastasis on Overall Survival in Men With Castration-Resistant Prostate Cancer. J Clin Oncol.

[10] Scher HI, Morris MJ, Stadler WM, Higano C, Basch E, Fizazi K (2016). Trial Design and Objectives for Castration-Resistant Prostate Cancer: Updated Recommendations From the Prostate Cancer Clinical Trials Working Group 3. J Clin Oncol.

[11] von Eyben FE, Picchio M, von Eyben R, Rhee H, Bauman G (2018). (68)Ga-Labeled Prostate-specific Membrane Antigen Ligand Positron Emission Tomography/Computed Tomography for Prostate Cancer: A Systematic Review and Meta-analysis. Eur Urol Focus.

[12] Perera M, Papa N, Roberts M, Williams M, Udovicich C, Vela I (2019). Gallium-68 Prostate-specific Membrane Antigen Positron Emission Tomography in Advanced Prostate Cancer-Updated Diagnostic Utility, Sensitivity, Specificity, and Distribution of Prostate-specific Membrane Antigen-avid Lesions: A Systematic Review and Meta-analysis. Eur Urol. 2019. in press, doi 10:1016/eururol.

[13] von Eyben FE, Bauman G, Paganelli G (2020). Re: Silke Gillessen, Gerhardt Attard, Tomasz M. Beer, et al. Management of Patients with Advanced Prostate Cancer: Report of the Advanced Prostate Cancer Consensus Conference 2019. Eur Urol.

